# Time and lexicographic preferences in the valuation of EQ-5D-Y with time trade-off methodology

**DOI:** 10.1007/s10198-022-01466-6

**Published:** 2022-05-21

**Authors:** Stefan A. Lipman, Liying Zhang, Koonal K. Shah, Arthur E. Attema

**Affiliations:** 1 Erasmus School of Health Policy & Management, Erasmus University Rotterdam, Rotterdam, The Netherlands; 2 National Institute for Health and Care Excellence, London, UK; 3 School of Health and Related Research, University of Sheffield, Sheffield, UK; 4 PHMR Ltd, London, UK

**Keywords:** Time trade-off, Health state valuation, EQ-5D-Y, Time preferences, Lexicographic preferences, Perspective, I10

## Abstract

In the valuation of EQ-5D-Y-3L, adult respondents are asked to complete composite time trade-off (cTTO) tasks for a 10-year-old child. Earlier work has shown that cTTO utilities elicited in such a child perspective are generally higher than when adults take their own perspective. We explore how differences in time preference in child and adult perspectives could explain this effect. Furthermore, as cTTO valuation in a child perspective involves explicit consideration of immediate death for a child, we also consider how cTTO utilities could be affected by decision-makers lexicographically avoiding death in children. We report the results of an experiment in which 219 respondents valued 5 health states in both adult and child perspectives with either a standard cTTO or a lead-time TTO only approach, in which immediate death is less focal. Time preferences were measured in both perspectives. Our results suggest that utilities were lower when lead-time TTO, rather than cTTO, was used. We find large heterogeneity in time preference in both perspectives, with predominantly negative time preference. The influence of time preferences on utilities, however, was small, and correcting for time preferences did not reduce differences between utilities elicited in both perspectives. Surprisingly, we found more evidence for differences in utilities between adult and child perspectives when lead-time TTO was used. Overall, these results suggest that time and lexicographic preferences affect time trade-off valuation in child and adult perspectives, but are not the explanation for differences between these perspectives. We discuss the implications of our findings for EQ-5D-Y-3L valuation.

**Supplementary Information:**

The online version contains supplementary material available at 10.1007/s10198-022-01466-6.

## Introduction

EQ-5D is a health-related utility instrument that is widely used to derive quality-adjusted life years (QALYs) in economic evaluation [12]. The regular three-level version of EQ-5D (EQ-5D-3L) relies primarily on self-report and was originally designed for individuals aged 16 years and older. The past decades have seen increasing demand for measuring QALYs in child and adolescent populations [19]. As a result, the child-friendly version, EQ-5D-Y-3L, was developed from the EQ-5D-3L in 2009 by adapting the wording to be more suitable for populations as young as 8 years old [14].

Earlier work has shown that this adapted wording influences the utility assigned to health states derived from EQ-5D-Y-3L, compared to health states derived from EQ-5D-3L, with which it shares a structure [18]. Hence, separate value sets are needed for EQ-5D-Y-3L. The EQ-5D-Y valuation protocol [38], as well as the first studies applying it to generate value sets, have been recently published [42, 45]. This protocol retains the methods used for the valuation of adult EQ-5D instruments: discrete choice experiments (DCE) and composite time trade-off (cTTO). However, compared to adult EQ-5D valuation, a critical change implemented in the EQ-5D-Y-3L protocol is the recommendation to use a child perspective; i.e., adult respondents, instead of valuing hypothetical health states for themselves, value them considering the life of a 10-year-old child (henceforth: child perspective).

Although the exact effect of using a child perspective may depend on the method and health states under consideration, a series of studies suggest the use of a child perspective affects EQ-5D-Y-3L valuation [13, 17, 18, 27, 33, 40, 44]. For cTTO, the available evidence suggests that EQ-5D-Y utilities elicited with a child perspective will likely be higher than when adults value the same health state for themselves (henceforth: adult perspective),^1^ which may be interpreted as adults considering the same health impairments less severe for children than for themselves [13, 18, 27, 44]. However, these differences in observed utilities may not reflect differences in the perceived severities of the health impairments, but rather how different perspectives affect the ways in which people consider the trade-offs between life duration and quality of life required in cTTO. In this study, we explore the effect of two of such non-severity-related preferences that could be different between adult and child perspectives: (i) time preferences and (ii) avoidance of immediate death.

Earlier work suggested trade-offs in cTTO depend on time preferences [9, 25]. It has been hypothesized that time preferences may differ between adult and child perspectives [28], as earlier work on financial decision-making [39, 54] has shown that individuals discount less (i.e., they have lower time preferences) when they decide for others. Considering that in a child perspective, individuals are asked to trade-off length and quality of life for someone other than themselves, it could be expected that such self-other differences in time preference also extend to child and adult perspectives.

Furthermore, in cTTO, the utilities of the health states equal to or better than dead (BTD) are measured with a different method than health states worse than dead (WTD). Often, and particularly in the protocol implemented for the valuation of EQ-5D-Y-3L [38], the transition from the conventional to lead-time TTO only occurs when respondents explicitly prefer immediate death to life in impaired health [36]. In other words, the method involves explicit consideration of immediate death to transit to the method used to value WTD states. It may be expected that individuals’ tendency to avoid immediate death differs between adult and child perspectives. For example, individuals may be more inclined to avoid immediate death from child perspectives than adult perspectives, because they feel unable or not legitimized to decide that someone else is better off dead than alive [40]. Moreover, when the alternative to immediate death is a life in severely impaired health, some individuals consider these health states BTD for short durations, but WTD for longer durations. This is referred to as maximum endurable time (MET, Sutherland et al. [48]). MET may differ between adult and child perspectives, for example because individuals expect 10-year-old children to be better able to cope with health problems [13]. Thus, respondents may be more inclined to avoid death for children, regardless of health state severity, which will affect EQ-5D-Y-3L valuation with a child perspective.

Hence, in this study, we measure time preferences using both adult and child perspectives by adapting the direct method developed by Attema et al. [4]. This method can be used to correct TTO for time preferences, i.e., to see to which degree removing the influence of time preferences can explain the difference in utilities elicited with adult and child perspectives. Second, we examine the role of avoidance of immediate death by employing two different TTO operationalisations. As a benchmark, we include the method recommended in the EQ-5D-Y-3L protocol, i.e., cTTO. We compare cTTO to the valuation of EQ-5D-Y-3L with lead-time TTO only, in which no preference for immediate death is needed to transit from BTD to WTD health state valuation.

In the next section of this paper, we provide the theoretical background for both TTO operationalisations, and indicate the importance of time preference. Next, we outline the experiment used to explore the influence of time preferences and avoidance of death, and present the results of the experiment. In the final section of the paper, we discuss these results.

## Theoretical background

In this study, we assume the general QALY model holds [31]. In this model, health profiles of the form (*T*,*Q*), with *T* denoting years and *Q *denoting health status, are evaluated as: 

1 $$V\left( {T,Q} \right) = L\left( T \right)U\left( Q \right).$$

Here, *L*(*T*) denotes the utility function for life duration and *U*(*Q*) is the utility of *Q* in each period. In practice, e.g., in the valuation of EQ-5D-Y-3L [38], the linear QALY model [37] is assumed to hold, which is a special case of the general QALY model with *L*(*T*) = *T*. As is usual, we let ~ , ≽$,$ and ≻ denote indifference, weak preference, and strict preference, respectively.

In the general QALY model, we assume the zero-condition holds, i.e., (0,*Q*_1_) ~ (0,*Q*_2_) for any Q [32]. In other words, individuals are indifferent between all health profiles that have durations of 0 (i.e., imply immediate death). With the zero condition, it can be shown that being dead yields a utility of 0 [41].

### cTTO in the general QALY model

The cTTO method used comprises two different TTO methods, here referred to as standard TTO and lead-time TTO [16]. In the standard TTO, we elicit an indifference of the form (10,*Q*) ~ (*X*,*FH*), with *FH* denoting full health. According to the general QALY model, this is evaluated by:

2 $$L\left( {10} \right)U(Q) \, = L\left( X \right)U(FH).$$

Given the usual scaling of *U*(*FH*) = 1, this yields:

3 $$U(Q) = \frac{{L\left( {\text{X}} \right)}}{{L\left( {10} \right)}}.$$

Standard TTO questions can only elicit *U*(*Q*)$\ge$ 0, i.e., utilities for states better than or equivalent to being dead. For states not preferred to being dead (i.e., WTD states), a lead-time TTO question is asked; i.e., we elicit the indifference (10,*FH*;10,*Q*) ~ (*X*,*FH*). Here, (10,*FH*;10,*Q*) denotes 10 years in full health followed by 10 years in *Q*. This is evaluated by:

4 $$L\left( {10} \right)U\left( {FH} \right) + (L\left( {20} \right) - L\left( {10} \right))U\left( Q \right) \, = L\left( X \right)U\left( {FH} \right).$$

Scaling *U*(*FH*) = 1 and rearranging gives:

5 $$U(Q) = \frac{{L\left( X \right) - L\left( {10} \right) }}{{L\left( {20} \right) - L\left( {10} \right)}}.$$

When a cTTO approach is used, as in EQ-5D-Y-3L valuation [38], standard TTO questions are used for BTD states and lead-time TTO for WTD states. As such, only indifferences (10,*FH*,10,*Q*) ~ (*X*,*FH*) with *X* $\le 10$ are elicited, yielding *U*(*Q*)$\le 0$. Earlier work, however, has applied lead-time TTO also for BTD states (e.g., Attema et al. [7]), as it can also accommodate *U*(*Q*) > 0 whenever X > 10. In this study, we compare both approaches, by comparing respondents’ TTO valuation adult and child perspectives with cTTO and lead-time only TTO (i.e., all indifferences are elicited as (10,*FH*,10,*Q*) ~ (*X*,*FH*) with X between 0 and 20).

### Time and lexicographic preferences in valuation of EQ-5D-Y-3L

As can be seen from Eqs. 3 and 5, both standard and lead-time TTO require information about *L*(*T*) to elicit *U*(*Q*) under the general QALY model. If, instead, the linear QALY model is assumed to hold, standard TTO indifferences yield *U*(Q) = *X*/10, and lead-time TTO indifferences can be simplified to *U*(*Q*) = (*T* − 10)/10. The linear QALY model only holds with zero time preference, that is, whenever individuals do not discount the utility of life duration, *L*(*T*) = *T*. Going forward, whenever we refer to uncorrected TTO utilities, these will be evaluated assuming zero time preference. Seeing as the general QALY model allows for time preferences, calculating *U*(Q) through Eqs. 3 and 5 will be referred to as correcting for time preference. However, as outlined in the Introduction, in the valuation of EQ-5D-Y-3L, individuals are asked to use a child perspective, and time preferences may depend on the perspective used for valuation. Furthermore, in cTTO tasks, individuals are asked to consider whether a child living for 10 years in some state *Q* is preferable to a child dying immediately. Although technically a violation of the general QALY model, individuals may avoid death for children lexicographically, i.e., their trade-offs involving immediate death are not continuous. This implies that *L*(*T*) is discontinuous at 0. As discussed in the introduction, such discontinuity may be more pronounced for children than for adults. For the lead-time only condition, discontinuity around *L*(*T*) = 0 would not affect EQ-5D-Y-3L valuation as much, as this would only occur for states for which respondents sacrifice all lifetime (including all lead-time), i.e., when *U*(*Q*) = − 1. Although we will not use such notation extensively, we may introduce notation, such that *Lc*(*T*) denotes the utility of *T* years from a child perspective and *La*(*T*) denotes the utility of *T* years from an adult perspective. The goal of this study is to disentangle the effect of time preference and lexicographic avoidance of death in the valuation of EQ-5D-Y-3L, by measuring *La*(*T*) and *Lc*(*T*), and comparing cTTO valuations to lead-time TTO valuations.

To our knowledge, this is the first study to empirically test for differences in time preferences in child and adult perspectives, and hence, we formulate no hypotheses about in which direction this difference could occur. Earlier work has compared various time trade-off operationalisations, in particular in the process leading up to uptake of cTTO in EQ-5D valuation. Although not completely comparable (these studies used a variant of time trade-off for the valuation of WTD states that required arbitrary rescaling), these studies provide some evidence of differences between lead-time TTO and cTTO for BTD states. In particular, lead-time TTO was found to yield lower utilities for BTD states [7]. This effect could be interpreted as evidence suggesting lexicographic avoidance of immediate death, as such avoidance would (ceteris paribus) decrease the proportion of health states considered equal to or worse than dead. Other studies, however, have suggested that the increased duration could explain this finding [8, 16], which may be partially controlled for by correcting for time preference. To our knowledge, this is also the first study comparing valuation with lead-time TTO and cTTO in a child perspective, so there is a current lack of evidence on how differences in time trade-off valuation of EQ-5D-Y-3L utilities in adult and child perspectives will be affected by the two methods.

## Methods

We conducted an experiment in which the influence of time and lexicographic preferences was explored in the valuation of EQ-5D-Y health states for both child and adult perspectives.

### Sample, procedure, and design

This experiment was conducted with a sample of 219 students. All data were collected between March and May 2021 in The Netherlands. Students were recruited through systems used for recruiting students for research participation for course credits (*n* = 42) or monetary rewards (*n* = 177).^2^ The sample consisted of 141 females and 78 males and had a mean age of 21.6 (SD = 3.1). Due to restrictions related to the ongoing COVID-19 pandemic, all data were collected online, through videotelephony software (i.e., Zoom, for a discussion of the benefits and drawbacks of this approach, see: Lipman [20]). Respondents entered a Zoom meeting and received a general instruction about the purpose of the experiment. Next, they were assigned to a break-out room. In this break-out room, each respondent watched a short instructional video, in which all elements of the TTO tasks and time preference measurement were introduced (see Online Supplements for an example video). Afterward, each respondent completed the EQ-5D-Y-3L questionnaire and reported demographics (i.e., age and sex). Next, respondents started the experiment, which lasted around 30 min. The experiment was hosted online, programmed in Shiny, and a link to a demo version is found in the Online Supplements. Note that after receiving the pre-recorded instructions, the experiment was set up, such that each respondent could complete the survey by themselves, without an interviewer. Two experimenters were available to answer questions that respondents raised.

The experiment was operationalised with a 2 (Health state block) × 2 (TTO operationalisation) × 2 (Perspective) mixed subjects design, with two between-subjects factors and one within-subjects factor. The *Health state block* factor was operationalised between-subjects, i.e., each respondent valued one block of 5 EQ-5D-Y-3L health states. The *TTO operationalisation* used for this valuation was also randomized between-subjects, with respondents either assigned to the cTTO condition or the lead-time only TTO (henceforth: LT-TTO) condition. *Perspective* was operationalised within-subjects, meaning that each respondent valued 5 EQ-5D-Y-3L health states from both adult and child perspectives and completed measurements of time preference for both perspectives. Hence, the experiment consisted of four parts: two TTO parts and two time preference measurement parts. The order in which these four parts were completed was randomized.

### Health states

All health states included in this study were drawn from the EQ-5D-Y-3L instrument [14, 53]. This instrument describes the quality of life with five domains: mobility, looking after yourself, doing usual activities, having pain or discomfort, and feeling worried, sad or unhappy. EQ-5D-Y-3L distinguishes between three levels of severity in each domain: ‘no problems’, ‘some problems’, and ‘a lot of problems’. Typically, health states are denoted by 5-digit codes with each number representing the severity of the relevant domain, e.g., code 12231 would refer to a health state with no problems with mobility, some problems with self-care, some problems with usual activities, a lot of pain or discomfort, and not feeling worried, sad or unhappy. For this study, we used two blocks of 5 health states, selected from the study by Kreimeier et al. [18]. Block 1 consisted of states 11121, 22222, 32211, 33323, and 33333, while Block 2 consisted of states 11112, 22222, 11312, 13311, and 33333. These health states were selected to capture all levels of problems on each dimension, as well as covering a wide range of severity, i.e., the health states selected were the same as in Lipman et al. [27].

### Time trade-off operationalisation

The operationalisation of TTO depended on the condition respondents were assigned. If respondents were assigned to the cTTO condition, TTO was operationalised similarly to how it is applied in EQ-5D valuation studies [35, 38]. For adult perspectives, respondents were asked to consider that they themselves live in the described health, while for child perspectives, instead, respondents were asked to consider that this state affects a 10-year-old child. In the LT-TTO condition, respondents only faced LT-TTO tasks, regardless of whether the state considered is WTD or BTD. As a result, respondents were no longer asked to choose between 10 years in some health state (for themselves or a child) and immediate death, i.e., this method should not be affected by lexicographic avoidance of immediate death. The LT-TTO condition is otherwise similar to the cTTO condition. A few changes were implemented in this experiment to facilitate self-completion. In both conditions, a bisection search procedure is implemented, to obtain TTO indifferences yielding utilities at a 0.5-year precision. This bisection choice procedure commenced at the mid-point of the scale used, i.e., in the cTTO condition, it asked respondents to choose between 10 years in impaired health and immediate death, whereas in the LT-TTO condition, respondents are asked to choose between 10 years in full health, or 10 years in full health *followed by* 10 years in impaired health.

### Measurement of time preferences

We will use the direct method to measure time preferences [4] on a domain between *L*(0) and *L*(20). The direct method lets a subject compare two simple health profiles with horizon *T* = 20, which are both combinations of two health states, e.g., FH and some imperfect state *Q* (in this study: chronic back pain).^3^ The difference between the profiles is that one starts with the better health state FH and ends with the worse state *Q*, whereas the other starts with *Q*, followed by an improvement toward FH. The transition from FH to *Q* or vice versa occurs at the same point in time in both profiles, e.g., at $T_{d1/2} .$ The purpose is to elicit the point $T_{d1/2}$, such that an individual is indifferent between the two profiles, that is:

6 $$\left( {T_{d1/2} , FH;\left( {20 - T_{d1/2} } \right),Q} \right)\sim \left( {T_{d1/2} , Q;\left( {20 - T_{d1/2} } \right),FH} \right).$$

According to the general QALY model, such an indifference is evaluated as follows:

7 $$L(T_{d1/2} )U\left( {FH} \right) \, + \, \left[ {L\left( {20} \right) - L(T_{d1/2} )} \right]U\left( Q \right) \, = L(T_{d1/2} )U\left( Q \right) \, + \left[ {L\left( {20} \right) - L(T_{d1/2} )} \right]U\left( {FH} \right).$$

Standardizing U(FH) = 1 and rearranging give:

8 $$L\left( {T_{d1/2} } \right)\left( {1 - U\left( Q \right)} \right) \, = \left[ {L\left( {20} \right) - L\left( {T_{d1/2} } \right)} \right]\left( {1 - U\left( Q \right)} \right).$$

From Eq. 2, it can be seen that (1 − *U*(Q)) cancels out, and hence, we know that *L*($$T_{d1/2}$$) = *L*(20) − *L*($$T_{d1/2} )$$. In other words, the period [0,$$T_{d1/2}$$] has the same utility as [$$T_{d1/2}$$,20]. Throughout, we will scale *L*(T), such that *L*(0) = 0 and *L*(20) = 1. Under this scaling, *L*($$T_{d1/2}$$) = *L*(20)- *L*($$T_{d1/2} )$$ implies *L*($$T_{d1/2}$$) = ½. Attema et al. [4] show how, depending on the number of elicitations, this method allows for a measurement of the utility function for life duration up to any desired amount of precision. For example, we can next find $$T_{d1/4}$$, such that *L*[0, $$T_{d1/4}$$] = *L*[ $$T_{d1/4}$$, $$T_{d1/2}$$] and, hence, *L*($$T_{d1/4}$$) = 1/4. The Direct Method was also operationalised in a 5-choice bisection search procedure, which allowed estimating indifferences at 0.5-year increments. 

The operationalisation furthermore depended on perspective. For the adult perspective, respondents were explained that they were choosing treatments that would alleviate chronic back pain they themselves experienced at different points in time. From the child perspective, respondents were instructed to imagine a 10-year-old child suffering from chronic back pain and they were asked to choose between treatments that would alleviate its health problems. Furthermore, we can characterize individuals’ discounting by estimating the area under the curve (AUC) of *L*(*T*). Under our scaling, the shape of $L\left( T \right){ }$ is concave [linear, convex] whenever ${\text{AUC }} > 0.5{ }[{\text{AUC }} = 0.5,{\text{ AUC }} < 0.5]$. To correct for time preference, as in Eqs. 3 and 5, we used linear interpolation to approximate when necessary, which allows for correcting cTTO utilities without assuming a parametric form for $L\left( T \right)$. We present a worked-out example in Box I.

Box I: Correcting EQ-5D-Y-3L for time preference: a worked-out example. Below we indicate how TTO utilities can be corrected for time preference elicited by our discounting task, and how $La(T)\,\ne\,Lc(T)$ could yield different *U*(Q). We do so separately for better than death (BTD) and worse than death (WTD) health states. Imagine for example, that we have elicited three points of the utility function for both adults and children, giving *La*(3) = 0.25, *La*(8) = 0.5, *La*(12) = 0.75, and *Lc*(5) = 0.25, *Lc*(10) = 0.5 and *Lc*(15) = 0.75. For this person, the utility of the next 8 years *for themselves* are equivalent to the 12 years that follow it (i.e. the direct method yielded *L*(${T}_{d1/2}=$ 8) = L(20)-*L*(${T}_{d1/2}=$ 8)). This person has positive time preference *for themselves*, as years further away in the future have less value than years closer to T = 0. For a child, however, their indifferences suggest that *Lc*(${T}_{d1/2}=$ 10) = *Lc*(20) − *Lc*(${T}_{d1/2}=$ 10), i.e. the first 10 years are worth the same as the next 10 years. This indicates that *for children* this person does not discount the future. Hence, we know that for this person, *Lc*(*T*) = T *for children.*

First, imagine our respondent completed a standard TTO question (i.e. for a state BTD) with *X* = 6 for both the child and adult perspective. To correct for time preferences, in order to solve Eq. 3 for the adult perspective, we need to find *La*(6) and *La*(10). *La*(6) is found by linearly interpolating between *La*(3) and *La*(8), giving 0.25 + (6–3)/(8–3) × 0.25 = 0.25 + 0.15 = 0.40. Similarly, we obtain *La*(10) = 0.5 + (10–8)/(12–8) × 0.25 = 0.625. Hence, *U*(*Q*) = 0.40/0.625 = 0.64. For a child, seeing as this respondent has zero time preference, we find the uncorrected utility by 6/10, i.e. 0.6. Hence, the fact that we found the same value of *X* for both perspectives was due to the downward pressure of time preference in adult perspective, which was absent in the child perspective.

Second, let us look at the case of a WTD state, where a lead-time TTO procedure has indicated that the respondent is indifferent between a health episode consisting of 10 years in FH followed by 10 years in health state *Q*, and an episode consisting of just living 7 years in FH (in both the adult and child perspective). Again, given that we assumed no discounting for a child, we can calculate the uncorrected utility as *U*(*Q*) = (7–10)/10 = − 0.3. For adults, we need to calculate the corrected utility by means of Eq. 5. We already determined that *La*(10) = 0.625 and we can compute *La*(7) to be 0.25 + (7–3)/(8–3) × 0.25 = 0.45. Therefore, we obtain *U*(*Q*) = (0.45–0.625)/(1–0.625) = − 0.467, which is lower than utility for a child. Finally, we can use the same approach when correcting lead-time TTO indifferences for states BTD. For example, imagine our respondent was indifferent between a health episode consisting of 10 years in FH followed by 10 years in health state Q, and an episode consisting of living 12 years in FH *in both perspectives*. For the child, the uncorrected utility for this state would be *U*(*Q*) = 12/10 = 0.2. The corrected utility for the adult perspective for our respondent would be: *U*(*Q*) = (0.75–0.625)/(1–0.625) = 0.333.

In these examples, we assumed throughout that individuals had positive time preference for adults and zero time preference for children. The effect of correcting for time preference in this case is opposite for WTD states than BTD states. This is a general principle that always occurs when comparing positive time preference to no time preference: the utility of BTD states is biased downward without correction, whereas the utility of WTD states is biased upwards without correction. Conversely, the opposite pattern holds when comparing negative time preference to zero time preference.

## Results

Seeing as our analyses were exploratory, we report all tests without correction for multiple hypothesis testing. Due to a technical error, far fewer respondents were assigned to Block 1 (*n* = 55) than to Block 2 (*n* = 164). This imbalance in our design means that for some health states, we have more observations than others.

### Data quality and response patterns per condition and perspective

Before reporting the main descriptive results of our study, we compare response patterns and data quality (adapted from Alava et al. [1] in the two conditions (by perspective), as shown in Table 1. The distribution of the TTO utilities per condition can be found in the Online Supplements. Overall, we find a slight difference in the amount of WTD states between the two conditions. That is, in the cTTO condition, we found *U*(*Q*) < 0 for 17% of all observations (17% in either perspective), whereas in the LT-TTO condition, we find 23% negative utilities (22% for adults, 24% for children). This difference was statistically significant (Chi-squared test, *p* < 0.001). Overall, data quality appeared sufficient, with very few strict violations of dominance occurring, across all possible violations. That is, the proportion of responses violating dominance was between 3.2 and 4.6%. The distribution of non-trading responses (i.e., *U*(*Q*) = 1) was not independent, suggesting that we observed more non-trading responses in the cTTO condition (Chi-squared, *p* < 0.006). Conversely, we observed more zero responses in the LT-TTO condition (Chi-squared, *p* < 0.009).

**Table 1:** Data quality per condition and perspective (numbers indicate amount of occurrences)

Response pattern	cTTO Adult	LT-TTO Adult	cTTO Child	LT-TTO Child
**Non-trading responses (*U*(*Q*) = 1)**	**48**	**21**	**45**	**33**
All-in trading responses (*U*(*Q*) = − 1)	20	20	9	15
**Zero responses (U(Q) = 0)**	**9**	**23**	**11**	**23**
Fewer than 3 out of 5 unique observations^a^	4	6	3	3
Respondents without negative utilities	48	35	44	41
Respondents without 0.5-year increments^b^	10	11	11	10
Weak violation of dominance for 33333 (e.g., *U*(22222) < = *U*(33333))	55	46	44	55
Strict dominance violation (e.g., *U*(22222) < *U*(33333))	22	21	29	31

^a^Numbers indicate the number of respondents who gave the same value to all 5 health states valued, or had only 2 unique valuations (e.g., valuing 5 states as: 0.5, 0.5, 0.75, 0.5, 0.75)

^b^Numbers indicate the number of respondents who only gave responses in full-year increments, i.e., for all states $U\left(Q\right)\in 1, 0.9, 0.8, 0.7, 0.6, 0.5, 0.4, 0.3, 0.2, 0.1, 0, -0.1, -0.2, -0.3, -0.4,-0.5, -0.6, -0.7,-0.8, -0.9. -1$

Bold-faced numbers indicate that Chi-squared tests were significant with *p* < 0.05

### Uncorrected EQ-5D-Y-3L utilities: descriptive results

Table 2 shows the uncorrected TTO utilities elicited in our experiment. With 1 exception (33323), we find evidence, suggesting that cTTO utilities are higher than LT-TTO utilities. This between-subjects effect was significant for 3 states in the adult perspective (11112, 11312, and 22222), as well as in the child perspective (11112, 13311, and 22222).

**Table 2:** Mean uncorrected TTO utilities (standard deviations) for all states per condition and perspective

State	Adult: cTTO	Adult: LT-TTO	Child: cTTO	Child: LT-TTO
Block 1	*n* = 25	*n* = 30	*n* = 25	*n* = 30
11121	0.82 (0.18)	0.78 (0.23)	0.82 (0.21)	0.81 (0.24)
32211	0.52 (0.39)	0.45 (0.49)	0.62 (0.29)	0.49 (0.48)
33323	– 0.22 (0.54)	– 0.09 (0.51)	– 0.11 (0.53)	– 0.08 (0.49)
Block 2	*n* = 86	*n* = 78	*n* = 86	*n* = 78
11112	**0.90 (0.14)**	**0.73 (0.32)**	**0.89 (0.14)**	0.79 (0.25)*
11312	**0.61 (0.37)**	**0.37 (0.44)**	0.58 (0.35)	0.48 (0.37)*
13311	0.46 (0.46)	0.37 (0.51)	0.61 (0.29)*	0.45 (0.48)*
Both blocks	*n* = 111	*n* = 108	*n* = 111	*n* = 108
22222	**0.58 (0.35)**	**0.4 (0.44)**	**0.61 (0.29)**	**0.43 (0.42)**
33333	– 0.15 (0.58)	– 0.25 (0.5)	– 0.14 (0.56)	– 0.17 (0.5)*

*Indicates that the within-subjects difference between adult and child valuation was significant (paired *t* test, *p* < 0.05)

Whenever TTO utilities are printed boldfaced, this indicates that cTTO utilities were significantly higher than LT-TTO utilities (*t* test, *p* < 0.05)

We observed considerable heterogeneity TTO utilities elicited in adult and child perspectives, in both conditions (see Online Supplements). Whenever significant differences were observed, these suggested that TTO utilities were lower in the adult perspective than in the child perspective. In fact, the average difference across all observations was − 0.048, i.e., about half a year extra was traded off for the same state for adults (see Online Supplements for the distribution of these differences). We also tested if such differences existed per state. In the cTTO condition, we find such evidence for state 13311 only, whereas in the LT-TTO condition, such evidence was observed for states 11112, 11312, 13311, and 33333.

### Time preference in both perspectives

**Fig. 1:**
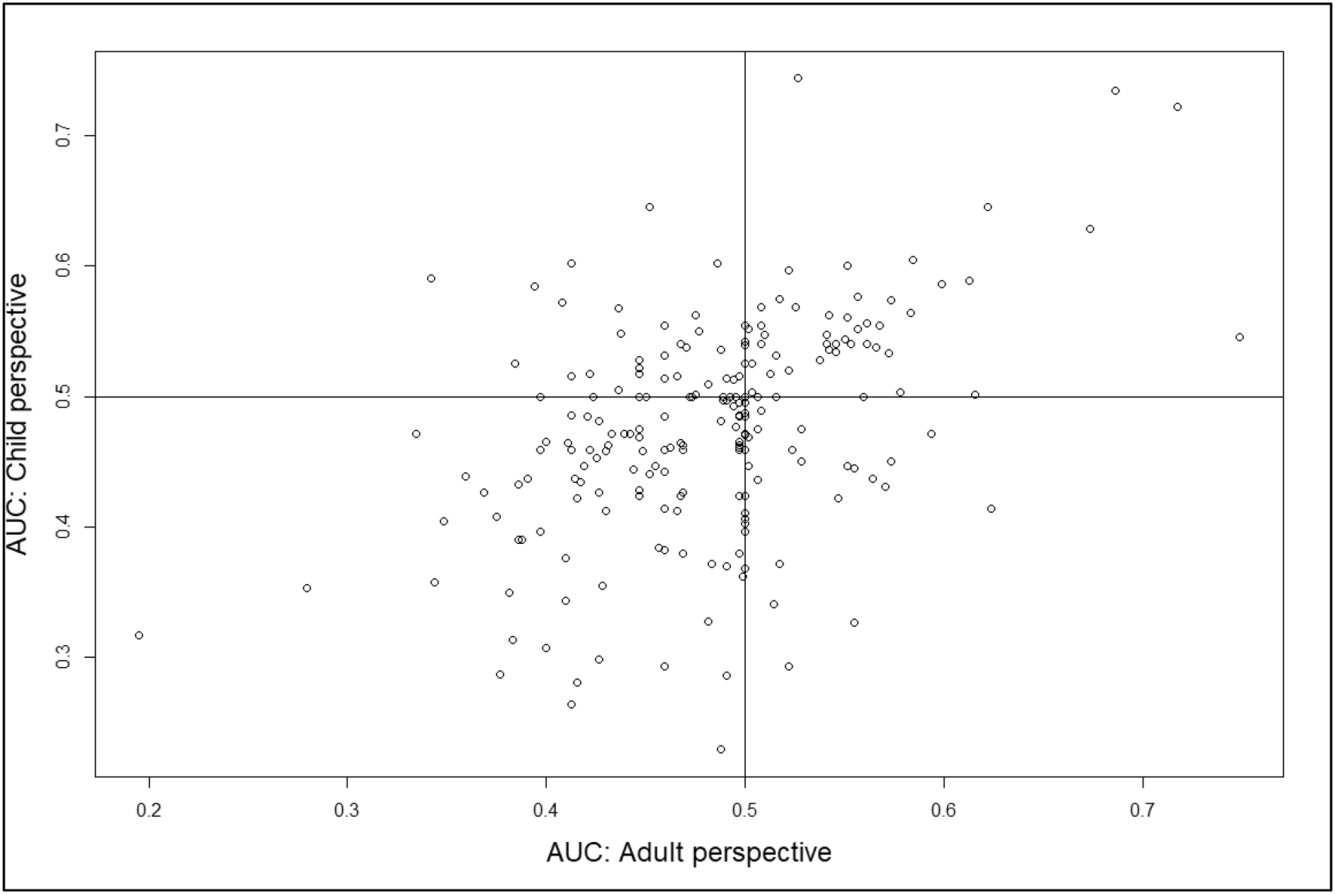
Scatterplot showing area-under-the-curve (AUC) data for adult and child perspective

Figure 1 plots the AUC for adult and child perspectives, with the classification of respondents presented in Table 3. We find no evidence for an overall difference in discounting between adult and child perspectives (paired Wilcox test, *p* = 0.66). That is, median AUC was 0.49 and 0.48 in adult and child perspectives respectively, suggesting a slight tendency toward negative time preference. However, Fig. 1 clearly shows that *La*(*T*)$\ne$ *Lc*(*T*), i.e., life duration for an adult and child is not discounted at the same rate for many individuals. Furthermore, AUCs in both perspectives are (weakly) positively correlated, Pearson’s *r* (217) = 0.454, *p* < 0.001), suggesting systematicity in time preferences across perspectives.

**Table 3:** Classification of area under the curve (AUC) for both perspectives

	AUC: adult		
AUC: Child	Negative discounting	No discounting	Positive discounting
Negative discounting	89	9	29
No discounting	15	4	4
Positive discounting	20	4	45

Such systematicity can also be seen in Table 3, which shows that the two most occurring classifications are negative time preference in both perspectives or positive time preference in both perspectives. It can, also, be concluded that regardless of the perspective used, respondents are least likely to have no time preference.

### TTO utilities after correction for time preference: descriptive results

Table 4 reports descriptive results for TTO utilities after correcting for time preference. As would be expected with almost no evidence for time preference at the aggregate level, correcting for time preference has little effect on the mean EQ-5D-Y-3L utilities. That is, 28 out of the 32 mean utilities reported in Table 4 are not significantly different from those reported in Table 2 (paired *t* tests, all *p*’s > 0.07). We only find evidence for differences after correction in 4 cases. First, the utility for state 11312 valued from a child perspective with LT-TTO only is significantly higher after correction (Paired *t* test, *p* = 0.04). Second, the utility for state 33323 valued from an adult perspective with the cTTO task is significantly lower after correction (paired *t* test, *p* = 0.049), and the same holds for state 33333 from both perspectives (LT-TTO condition, paired *t* test, *p*’s < 0.04). In line with this non-systematic effect of correction, those states for which significant differences existed between the cTTO and LT-TTO conditions remained significant after correction (i.e., 11112, 11312 for adult perspectives, 13311 for child perspectives, and 22222, see Table 4). Differences between the valuation of EQ-5D-Y utilities with adult and child perspectives were also unaffected by correction for time preference. The mean difference across all observations was -0.045, i.e., a reduction of 0.003 compared to uncorrected EQ-5D-Y-3L utilities (see Online Supplements for the distribution of these differences). When we compared the difference between EQ-5D-Y-3L utilities elicited with adult or child perspectives per health state, we found no evidence for a reduction in difference for 15 out 16 comparisons (paired t test, all *p*’s > 0.09), with state 33,323 in the LT-TTO condition only as a single exception (paired *t* test, *p* = 0.04).

**Table 4:** Mean corrected TTO utilities (standard deviations) for all states per condition and perspective

State	Adult: cTTO	Adult: LT-TTO	Child: cTTO	Child: LT-TTO
Block 1	*n* = 25	*n* = 30	*n* = 25	*n* = 30
11121	0.82 (0.18)	0.79 (0.23)	0.82 (0.21)	0.82 (0.24)
32211	0.52 (0.41)	0.44 (0.55)	0.61 (0.3)	0.51 (0.5)
33323	– 0.29 (0.63)	– 0.15 (0.64)	– 0.2 (0.68)	– 0.14 (0.57)
Block 2	*n* = 86	*n* = 78	*n* = 86	*n* = 78
11112	**0.9 (0.13)**	**0.73 (0.34)**	**0.89 (0.16)**	0.81 (0.26)*
11312	**0.62 (0.36)**	**0.33 (0.67)**	0.52 (0.73)	0.51 (0.39)*
13311	0.47 (0.48)	0.32 (0.75)	0.61 (0.37)*	0.45 (0.58)*
Both blocks	*n* = 111	*n* = 108	*n* = 111	*n* = 108
22222	**0.58 (0.37)**	**0.36 (0.67)**	**0.62 (0.31)**	**0.43 (0.49)**
33333	– 0.16 (0.63)	– 0.34 (0.75)	– 0.25 (0.95)	– 0.17 (0.74)*

*Indicates that the within-subjects difference between adult and child valuation was significant (paired *t* test, *p* < 0.05)

Whenever TTO utilities are printed boldfaced, this indicates corrected cTTO utilities were significantly higher than corrected LT-TTO utilities (*t* test, *p* < 0.05)

### Regression results

These descriptive results were substantiated with a set of regression analyses of the elicited EQ-5D-3L-Y utilities, reported in Table 5. All regression models were specified with subject random effects, and fixed effects for: (i) condition—which has value 0 for cTTO valuation and 1 for LT-TTO valuation, (ii) perspective—which has value 0 for adult perspective and 1 for child perspective), and (iii) health states—with health state severity controlled for through a set of dummies with state 11121 as reference. All coefficients are estimated with maximum likelihood.

**Table 5:** Mixed-effects regression results for corrected and uncorrected EQ-5D-Y-3L utilities

			Uncorrected		Corrected	
	(1)	(2)	(3)	(4)	(5)	(6)
Condition	– 0.120*** (0.0432)	– 0.149*** (0.0496)	– 0.118*** (0.0384)	– 0.132*** (0.0438)	– 0.122** (0.0499)	– 0.166*** (0.0583)
Perspective	0.0468*** (0.0174)	0.0184 (0.0232)	0.0482*** (0.0144)	0.0343* (0.0198)	0.0455** (0.0229)	0.00240 (0.0302)
Correction	– 0.0224** (0.0109)	– 0.0224** (0.0109)				
Perspective × Condition		0.0577* (0.0346)		0.0281 (0.0288)		0.0874* (0.0456)
Heath state						
11112	0.0196 (0.0515)	0.0196 (0.0515)	0.0124 (0.0424)	0.0124 (0.0424)	0.0250 (0.0503)	0.0250 (0.0504)
22222	– 0.309*** (0.0450)	– 0.309*** (0.0450)	– 0.308*** (0.0391)	– 0.308*** (0.0391)	– 0.310*** (0.0445)	– 0.310*** (0.0445)
32211	– 0.294*** (0.0489)	– 0.294*** (0.0489)	– 0.291*** (0.0477)	– 0.291*** (0.0477)	– 0.297*** (0.0504)	– 0.297*** (0.0504)
11312	– 0.304*** (0.0497)	– 0.304*** (0.0497)	– 0.302*** (0.0424)	– 0.302*** (0.0424)	– 0.309*** (0.0484)	– 0.309*** (0.0485)
33323	– 0.966*** (0.0721)	– 0.966*** (0.0721)	– 0.931*** (0.0658)	– 0.931*** (0.0658)	– 1.000*** (0.0799)	– 1.000*** (0.0799)
13311	– 0.341*** (0.0520)	– 0.341*** (0.0520)	– 0.339*** (0.0451)	– 0.339*** (0.0451)	– 0.345*** (0.0507)	– 0.345*** (0.0507)
33333	– 1.026*** (0.0516)	– 1.026*** (0.0516)	– 0.991*** (0.0448)	– 0.991*** (0.0448)	– 1.063*** (0.0543)	– 1.063*** (0.0543)
Constant	0.858*** (0.0470)	0.872*** (0.0486)	0.848*** (0.0410)	0.855*** (0.0425)	0.847*** (0.0461)	0.869*** (0.0471)
*N*	4380	4380	2190	2190	2190	2190

Standard errors in parentheses. All standard errors are clustered at the individual level

*p < 0.10, **p < 0.05, ***p < 0.01

Condition = 0: cTTO, Condition = 1: LT-TTO only. Perspective = 0: adult perspective and Perspective = 1: child perspective. Correction = 1: after correction. We have eight health states in total; here, we take the mild state 11121 as the reference when interpreting the change of the TTO utility for each health state

Multiple model specifications were used. Model 1 and 2 (i.e., columns 1 and 2) were run on all EQ-5D-Y-3L utilities, both before and after correction. Therefore, models 1 and 2 also include a fixed effect for ‘Correction’, which is a dummy taking value 0 for uncorrected EQ-5D-Y-3L utilities and 1 for these utilities after correction. Model 1 shows that EQ-5D-Y-3L utilities were significantly lower after correction for time preference. To have a more direct comparison of the role time and lexicographic preferences, we ran separate models for uncorrected (Models 3 and 4) and corrected (Models 5 and 6) EQ-5D-Y-3L. Models 2, 4, and 6 include interaction effects between the valuation method (cTTO or LT-TTO) and perspective. Several additional specifications, demonstrating the robustness of our main conclusion, e.g., including demographics or order effects, are reported in the Online Supplements.

The effects of health states can be interpreted as decremental utility compared to 11121. As expected, across all model specifications, almost all the coefficients are negative (i.e. more disutility) and significant, except 11112 for which disutility is similar to 11121. The effect of the condition is also negative across all models, suggesting that LT-TTO yields lower utilities. For example, Models 1, 3, and 5 suggest that individuals are willing to give up around 1.2 years more using LT-TTO compared to cTTO. Furthermore, EQ-5D-Y-3L utilities were generally higher with a child perspective compared to an adult perspective, although the difference is small (e.g., around 0.47 years in Model 1).

After including an interaction between condition and perspective in Model 2, we found a significant, positive coefficient for the interaction, which implies that the positive discrepancy between child and adult perspectives is larger in the lead-time TTO condition. Besides, the coefficient of the perspective is insignificant in Model 2, suggesting that the difference between adult and child is not significant in the cTTO valuation.

Looking at Models 4 and 6 before and after the correction of time preference, only for Model 6 (i.e., corrected EQ-5D-Y-3L utilities), we found a significant, positive coefficient for the interaction between condition and perspective, which implies that the positive discrepancy between child and adult perspectives is larger in the lead-time TTO condition after correction. Note, however, that in this model, the perspective main effect is not significant, which could suggest that the difference between adult and child perspectives was only apparent after correction in the LT-TTO condition (and not for the cTTO valuation).

## Discussion

Health state valuations obtained for the EQ-5D-Y-3L, valued with a child perspective, are generally higher than those obtained for the adult version of the EQ-5D (valued with an adult perspective). This study has explored to what degree this difference can be attributed to the time and lexicographic preferences. In our study, respondents valued EQ-5D-Y-3L from both adult and child perspectives, using either cTTO or only lead-time TTO methods. We separately measured time preferences using the direct method [4], again from both perspectives. Several surprising findings emerged from our study.

First, we found considerable heterogeneity in time preference, with the median respondent only slightly deviating from zero discounting (i.e., no time preference). In line with this finding, perhaps unsurprisingly, correcting TTO valuation for time preference had little to no effect on EQ-5D-Y valuation, neither for composite TTO nor for lead-time TTO. The absence of time preferences for health outcomes has been reported before [22, 50], although there is also a substantial number of studies finding positive time preferences for health [3, 4, 52]. Surprisingly, the modal preference across both perspectives was negative time preference, i.e., a preference for being healthy in the future. Negative time preference is typically not accounted for in constant discounting models [43], but it has been found to be prevalent in health preference research [21, 23, 29, 50], potentially because of anticipation or dread with health impairments and improvements in the future [30, 50]. Hence, our work provides more evidence that correcting for time preference in EQ-5D valuation requires methods that can accommodate negative time preference.

Second, we find no overall evidence for different time preferences in adult and child perspectives. As such, our results suggest that child life duration is not discounted at a different rate than adult life duration, on average. Combined with the only slightly negative time preference, this suggests that on average, the assumption of no time preferences across adult and child perspectives is relatively accurate. However, our study shows that this assumption is very unlikely to hold at the individual level, as only a small minority *actually* satisfies zero discounting or equal time preference in adult and child perspectives. This suggests that approaches to correcting time preferences may require individual level correction as argued in other work [24, 25]. It is important to mention, however, that our conclusions about the (lack of) effects of correcting for time preference, as well as the need for individual-level correction, assume that time preferences can be reliably measured (with the direct method). Only a few studies have studied test–retest reliability of the direct method: correlations between initial and repeated measures ranged between 0.74 [4] and 0.89 [6]. Future work should explore the reliability of the direct method further, as well as determine what level of reliability is sufficient.

Third, as in earlier work [13, 18, 44], we find some evidence for differences between EQ-5D-Y-3L valuation in adult and child perspectives. However, it appears that the effect of perspective is small and can differ between health states and individuals, as can also be concluded from earlier work on the influence of perspective in EQ-5D-Y-3L valuation [18, 27]. It is also surprising that we find no evidence for different valuations of state 33333, for which Shah et al. [44] found large differences in valuation. One explanation for small differences in utilities between perspectives, compared to Shah et al. [44], might lie in the student sample we used. That is, this young subject pool might have regarded the durations used in the TTO to be shorter than what they could reasonably expect and, hence, they were less willing to trade off any more years. However, earlier studies applying TTO in students found that students often were willing to trade even more years than respondents in a general public sample [25, 26]. A related explanation could be that our respondents were only approximately 10 years older than a 10-year-old child. In fact, our student sample is likely closer in age to a 10-year-old child than to the average age of the adult general public. Therefore, one might expect that the effect of moving from adult to child perspectives is relatively small. Yet, such a transition involves more than just considering health for someone younger, it also involves trading off life duration for another person. Lipman et al. [27], in a similar population, observed that it is particularly this change from self-oriented to other-oriented decision-making that explains lower EQ-5D-Y-3L utilities. Another important implication of using a student sample is that students are unlikely to be parents. Earlier work has found that parental status affects EQ-5D valuation [15], and EQ-5D-Y valuation with child perspectives in particular [40].

Fourth, we found some evidence that could suggest an effect of lexicographic avoidance of immediate death in cTTO. Our study compared cTTO valuation to LT-TTO valuation, and as the former method requires explicit preference for immediate death to value WTD health states, while the latter does not, differences between both methods may allow us to infer the effect of lexicographic preferences and their implications on EQ-5D-Y-3L utilities. However, as differences between cTTO and LT-TTO valuation can have various causes, which need not be related to avoidance of death [7, 8, 16, 51], our findings may have alternative explanations that require subsequent consideration. Our results suggest that the use of LT-TTO may avoid lexicographic preferences, as it produces lower utilities and more responses indicating utilities of 0. Both findings suggest that respondents, when a preference for immediate death is not required, are more inclined to respond in a way that states are considered equal to or worse than dead. Notably, this effect persisted after correcting for time preference, which shows that the effect was not related to the increased duration in LT-TTO, i.e., the violations of constant proportionality often observed cannot explain this effect [5, 10]. Nonetheless, LT-TTO yielded a larger difference between adult and child perspectives, i.e., one could conclude that lexicographic avoidance of death does not provide an explanation for differences between adult and child perspectives. If anything, our results suggest that the use of methods that reduce the potential censoring resulting from lexicographic preferences (e.g., LT-TTO) would increase differences between adult and child perspectives.

Besides requiring replication in future research, at least two alternative explanations related to other differences between cTTO and LT-TTO deserve mentioning. First, LT-TTO utilities could be lower than cTTO utilities for BTD states as respondents could answer using proportional heuristics [7, 46]. This heuristic, i.e., decision-making shortcut, implies that respondents may be inclined to trade off a stable proportion of their available life duration, regardless of how long that duration is. In LT-TTO trading off 25% of the maximum duration would yield lower utilities for BTD states than in cTTO (i.e. 15 out of 20 years and 7.5 out of 10 years). Importantly, this heuristic is not related to avoidance of death and is also not controlled for by correcting for time preference. Second, it has been suggested that LT-TTO valuation is affected by sequence effects [11], i.e., additive separability as assumed in the QALY model is violated. Generally, sequence effects refer to the observation that individuals prefer sequences that are improving over time [2]. LT-TTO involves a choice between a constant profile and the opposite of an improving sequence, i.e., a health profile with a reduction in health status after 10 years. Although sequence effects are particularly relevant when comparing LT-TTO to methods that involve lag-time [11, 51], they may also explain the differences observed between cTTO and LT-TTO in our study. In particular, the sequence effect suggests that fewer discounted QALYs are obtained in a profile in which full health is followed by impaired health, and as such, fewer years in full health would be considered equivalent to that profile (i.e., lower utilities).^4^ Note that sequence effects would also influence the direct method [4].

Importantly, our results provide no evidence that suggests that heterogeneity in EQ-5D-Y-3L utilities is related to differences in lexicographic preferences in adult and child perspectives. That is, we found that differences between EQ-5D-Y-3L utilities elicited in child and adult perspectives were more pronounced for lead-time only TTO valuation, i.e., in a method not affected by avoidance of immediate death. Our regression analyses even suggest that, after correcting for time preference, the difference in utilities between adult and child perspectives is only significant for lead-time only TTO valuation (and not in cTTO valuation). This suggests that the use of methods that avoid explicit consideration of death would not reduce differences between utilities elicited from adult and child perspectives.

The study reported in this paper does not come without limitations, which could provide additional explanations for differences between our study and earlier work. First, the use of a student sample limits the external validity of the results, as students are generally younger, healthier, and higher educated than the general public. Second, because of COVID-19, the experiment had to be administered by means of video software (without personal interviewers), which perhaps had led to reduced data quality compared to personal interviews [20, 34]. However, our analysis of data quality suggests that violations of dominance were rare (i.e., 3–5% of responses), and appeared to occur even less frequently than in some studies using personal interviews and/or an extensive quality control process. Our study also used a bisection elicitation procedure, rather than the search procedure used for data collection for EQ-5D value sets [47], which may be considered a limitation. Further limitations of the study are the imbalanced assignment of health state blocks, as well as some respondents being paid a financial reward and other respondents being awarded course credits. Although our main conclusions appeared to be unaffected by these limitations, future work should aim to avoid them.

The results make clear that correcting for time preferences and employing a lead-time TTO procedure instead of the composite TTO are no panacea for removing the systematic difference between adult and child health state utilities. Instead, the findings suggest that the different perspective used does not, or at most only partially, explain the reduced willingness to sacrifice life years in EQ-5D-Y. Hence, further research investigating the drivers of this phenomenon is warranted.

## Supplementary Information

Below is the link to the electronic supplementary material.

Supplementary file1 (DOCX 131 kb)

## Footnotes

1 Although in this study, the perspectives are referred to as child and adult perspectives, other authors have used different terminology or proposed taxonomies that could be used (e.g., Tsuchiya and Watson [49]). For example, Lipman et al. [23, 27] refer to the perspectives used here as: self-adult and proxy-child.

2 Our analyses indicated that there were no significant differences for any of the outcomes between these two groups (*T* test, all *p*’s > 0.05). Hence, any analysis is reported for the full sample. We also control for this difference in a set of regression analysis reported in Online Supplements.

3 Earlier work suggested that the choice of BTD state selected does not affect time preference, i.e., Attema and Brouwer [6] found similar estimates when comparing direct method estimates when using two BTD states to elicit time preferences, but differences occurred when a state was considered WTD.

4 To illustrate, imagine someone with *L*(*T*) = T, and assume *U*(*Q*) = 0.5. She would generate 15 QALYs from (10,FH; 10,*Q*). In LT-TTO, she would find this equivalent to 15 years in full health. If, however, due to the sequence effect the 10 years in Q are considered 20% less valuable, because they represent deterioration, the profile would only be worth 14 QALYs and thus equivalent to 14 years in full health. In cTTO, no sequences occur for states BTD, i.e., no sequence effects are present for BTD cTTO utilities.
