# Correction to: Time and lexicographic preferences in the valuation of EQ-5D-Y with time trade-off methodology

**DOI:** 10.1007/s10198-022-01530-1

**Published:** 2022-10-05

**Authors:** Stefan A. Lipman, Liying Zhang, Koonal K. Shah, Arthur E. Attema

**Affiliations:** 1 Erasmus School of Health Policy & Management, Erasmus University Rotterdam, Rotterdam, The Netherlands; 2 National Institute for Health and Care Excellence, London, UK; 3 School of Health and Related Research, University of Sheffield, Sheffield, UK; 4 PHMR Ltd, London, UK

## Correction to: The European Journal of Health Economics 10.1007/s10198-022-01466-6

The original article that was published contains errors in the abstract and result section head. Please find the corrected text with section heading.

## Abstract

In valuation of EQ-5D-Y-3L, adult respondents are asked to complete composite time trade-off (cTTO) tasks for a 10-year-old child. Earlier work has shown that cTTO utilities elicited in such a child perspective are generally higher than when adults take their own perspective. We explore how differences in time preference in child and adult perspectives could explain this effect. Furthermore, as cTTO valuation in a child perspective involves explicit consideration of immediate death for a child, we also consider how cTTO utilities could be affected by decision-makers lexicographically avoiding death in children. We report the results of an experiment in which 219 respondents valued 5 health states in both adult and child perspectives with either a standard cTTO or a lead-time TTO-only approach, in which immediate death is less focal. Time preferences were measured in both perspectives. Our results suggest that utilities were lower when lead-time TTO, rather than cTTO, was used. We find large heterogeneity in time preference in both perspectives, with predominantly positive time preference. The influence of time preferences on utilities, however, was small and correcting for time preferences did not reduce differences between utilities elicited in both perspectives. Surprisingly, we found more evidence for differences in utilities between adult and child perspectives when lead-time TTO was used. Overall, these results suggest that time and lexicographic preferences affect time trade-off valuation in child and adult perspectives, but are not the explanation for differences between these perspectives. We discuss the implications of our findings for EQ-5D-Y-3L valuation.

## Results

### Time preference in both perspectives

Figure 1 plots the AUC for adult and child perspectives, with the classification of respondents presented in Table 3. We find no evidence for an overall difference in discounting between adult and child perspectives (paired Wilcoxon test, *p* = 0.66). That is, median AUC was 0.51 and 0.52 in adult and child perspectives, respectively, suggesting a slight tendency towards positive time preference. However, Fig. 1 clearly shows that *La*(*T*) $\ne$ *Lc*(*T*), i.e. life duration for an adult and child is not discounted at the same rate for many individuals. Furthermore, AUCs in both perspectives are (weakly) positively correlated, Pearson’s *r* (217) = 0.454, *p* < 0.001), suggesting systematicity in time preferences across perspectives.

**Fig. 1:**
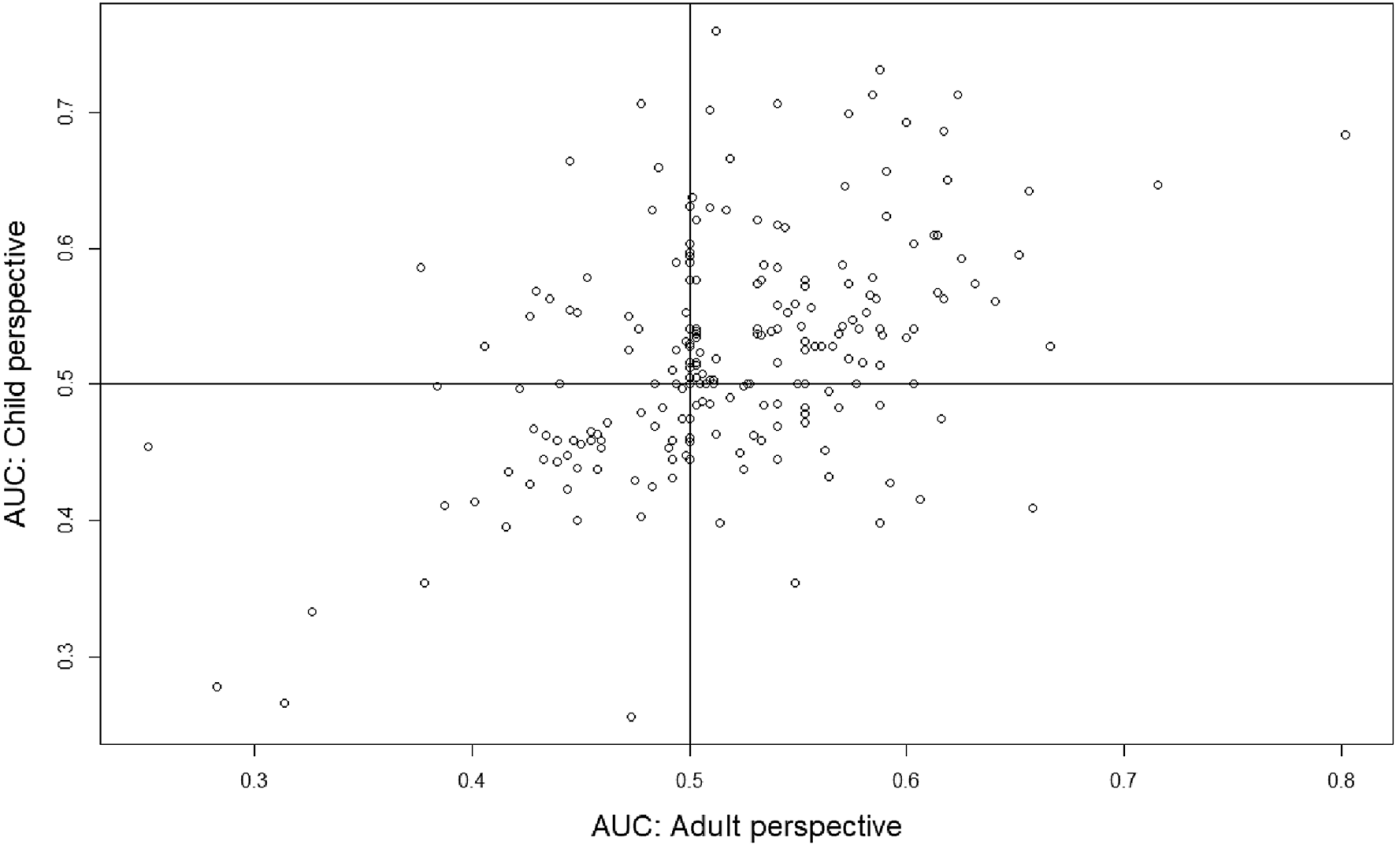
Scatterplot showing area-under-the-curve (AUC) data for adult and child perspective

**Table 3:** Classification of area-under-the-curve (AUC) for both perspectives

	AUC: adult		
AUC: child	Negative discounting	No discounting	Positive discounting
Negative discounting	46	4	20
No discounting	3	4	15
Positive discounting	29	9	89

Such systematicity can also be seen from Table 3, which shows that the two most occurring classifications are negative time preference in both perspectives or positive time preference in both perspectives. It can, also, be concluded that regardless of the perspective used, respondents are least likely to have no time preference.

## Discussion

First, we found considerable heterogeneity in time preference, with the median respondent only slightly deviating from zero discounting (i.e. no time preference). In line with this finding, perhaps unsurprisingly, correcting TTO valuation for time preference did not significantly alter EQ-5D-Y valuation, neither for composite TTO nor for lead-time TTO. Absence of time preferences for health outcomes has been reported before [5, 12], although there is also a substantial number of studies finding positive time preferences for health [2, 3, 13]. The modal preference across both perspectives was positive time preference, i.e. a preference for being healthy in sooner. We do find a considerable amount of negative discounting. Negative time preference is typically not accounted for in constant discounting models [11], but it has been found to be prevalent in health preference research [4, 8, 9, 12], potentially because of anticipation or dread with health impairments and improvements in the future [10, 12]. Hence, our work provides more evidence that correcting for time preference in EQ-5D valuation requires methods that can accommodate negative time preference. Second, we find no overall evidence for different time preferences in adult and child perspectives. As such, our results suggest that child life duration is not discounted at a different rate than adult life duration, on average. Combined with the only slightly positive time preference, this suggests that on average the assumption of no time preferences across adult and child perspectives is relatively accurate. However, our study shows that this assumption is very unlikely to hold at the individual level, as only a small minority actually satisfies zero discounting or equal time preference in adult and child perspectives. This suggests that approaches to correcting for time preferences may require individual level correction as argued for in other work [6, 7]. It is important to mention, however, that our conclusions about the (lack of) effects of correcting for time preference, as well as the need for individual-level correction, assume that time preferences can be reliably measured (with the direct method). Only a few studies have studied test-retest reliability of the direct method: correlations between initial and repeated measures ranged between 0.74 [2] and 0.89 [1]. Future work should explore the reliability of the direct method further, as well as determine what level of reliability is sufficient.
