# Supplementary material for: Time and lexicographic preferences in the valuation of EQ-5D-Y with time trade-off methodology
Source: Eur J Health Econ. 2022 May 21;24(2):293–305. doi: 10.1007/s10198-022-01466-6 (PMC9123877; doi:10.1007/s10198-022-01466-6)
Supplement: Supplementary file 1 — Supplementary file1 (DOCX 131 kb) [file 10198_2022_1466_MOESM1_ESM.docx]

# Online Supplements

These online supplements are included to the manuscript to provide additional methodological details as well as present additional results. In particular, we include the following Appendices in this Online Supplement:

- Appendix A: Link to instructional videos implemented in the task + Demo version of the task
- Appendix B: Additional figures depicting the distributions of our descriptive results.
- Appendix C: Additional regression analyses demonstrating the robustness of our finding

## Appendix A: Link to instructional videos implemented in the task + Demo version of the task

Interested readers can find the complete pre-recorded instructions for both conditions in the URLs provided below. Respondents would view this video after being assigned a break-out room and could ask questions at any point in time. Both videos include an instruction that was used to show the method included for measuring time preference (i.e. the direct method, Attema et al. (2012)). In Video 1 this part of the instruction starts around 4:30, whilst in Video 2 this starts at 4:05.

Video 1: cTTO instruction: <https://vimeo.com/566089626>

Video 2: LT-TTO instruction: <https://vimeo.com/566090182>

The authors have also prepared an online demo-version of the task for interested readers. The version used for data collection functioned differently, i.e. it did not allow for selection the condition or order, these were randomized. Also, for simplicity this demo version includes just 1 block of health states. This demo version of the task does not store any data.

Demo version: <https://referencepoints.shinyapps.io/DemoTaskTimeLexico/>

## Appendix B: Additional figures depicting the distributions of our descriptive results.

In this Appendix we present a series of figures that provide additional insight into the distribution underlying our main descriptive results. In particular, Figure B1 shows the overall distribution of uncorrected EQ-5D-Y-3L utilities in both perspectives, whereas Figure B2 shows these distributions by perspective.


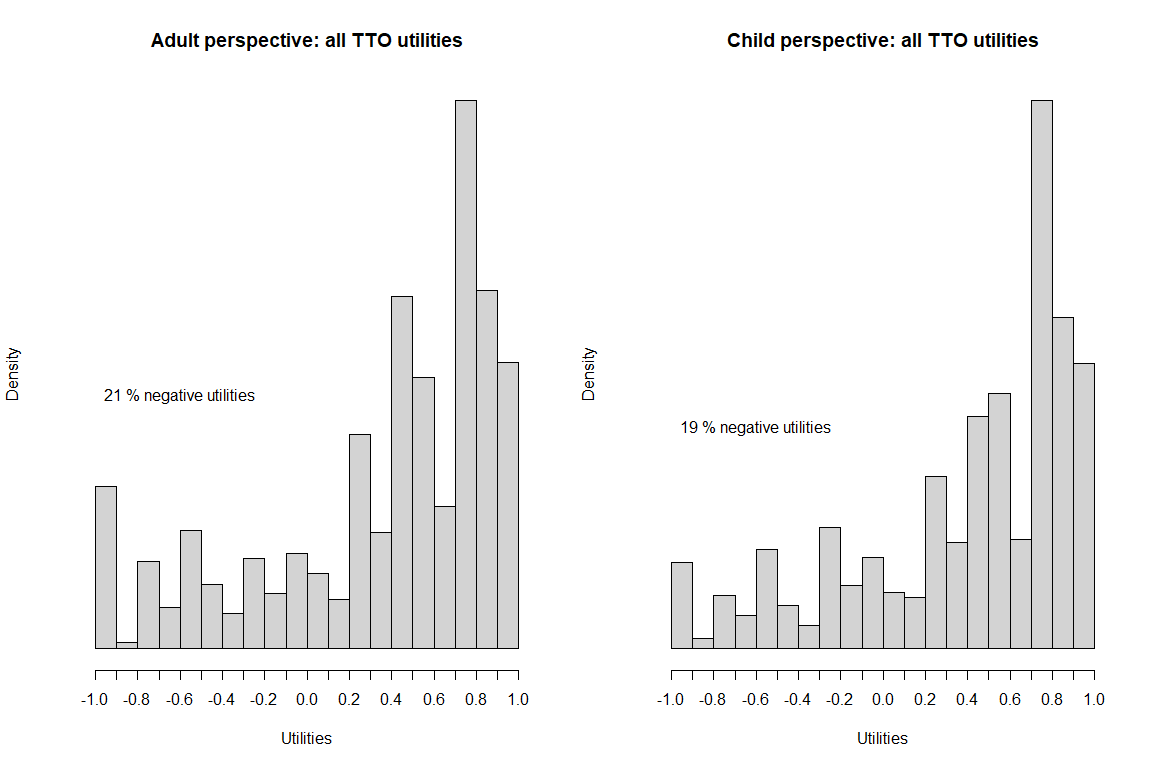
 Figure B1: Distribution of uncorrected EQ-5D-Y-3L utilities in adult and child perspectives


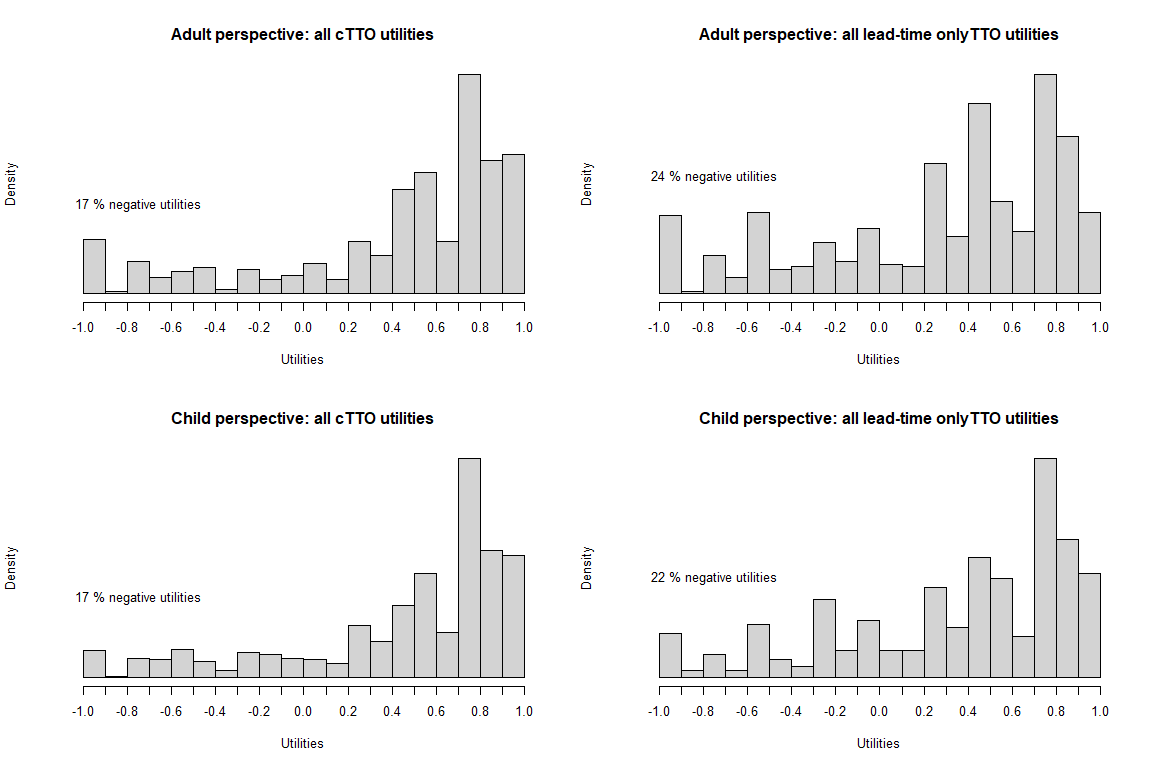


Figure B2: Distribution of uncorrected EQ-5D-Y-3L utilities per condition and perspective

In Figure B3, we show a Bland-Altman-plot showing the distribution of difference between EQ-5D-Y-3L utilities elicited with an adult or child perspective. This Bland-Altman-plot plots the difference between adult and child perspectives against their mean, which allows investigating if the difference depends on severity. The plot shows large heterogeneity, and if anything, this heterogeneity has a U shape, i.e. it is largest in the middle of the scale. This may suggest the occurrence of ceiling/floor effects and/or noise. In Figure B4, we show that this pattern occurs regardless of the condition used (i.e. cTTO or LT-TTO).


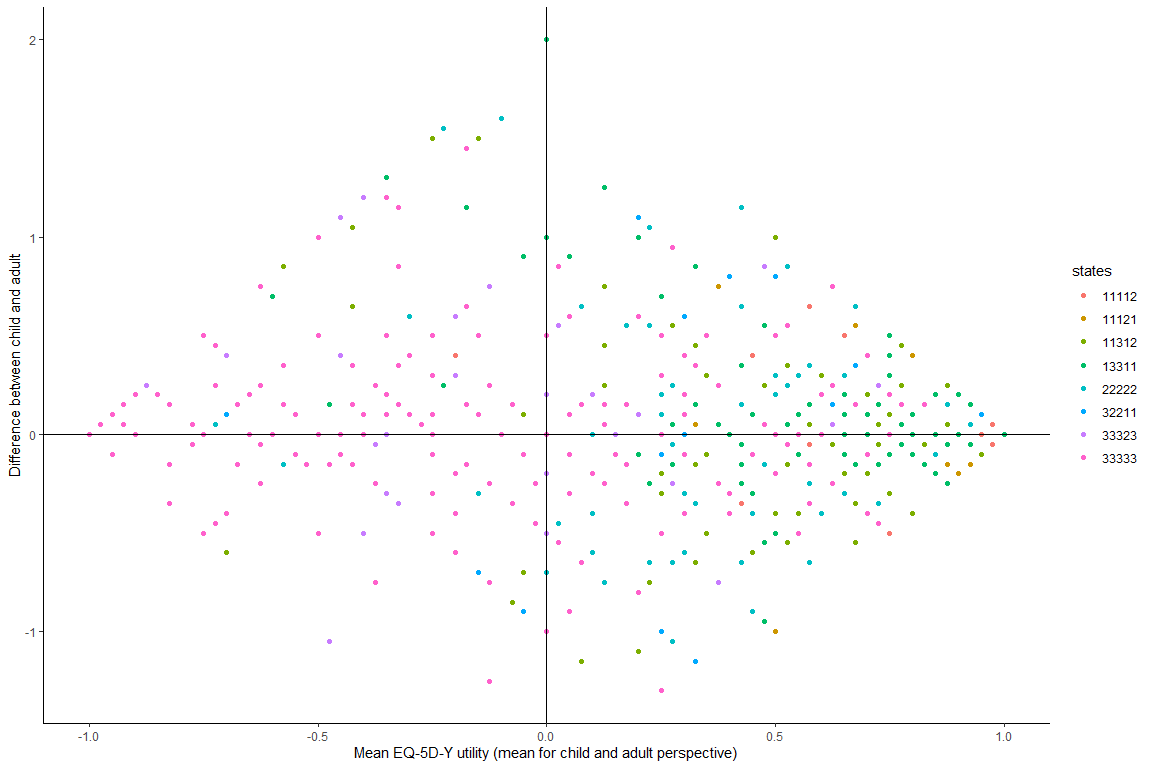


*Figure B3:* Bland-Altman plot with mean uncorrected EQ-5D-Y utility across child and adult perspectives plotted against the difference between the two perspectives.


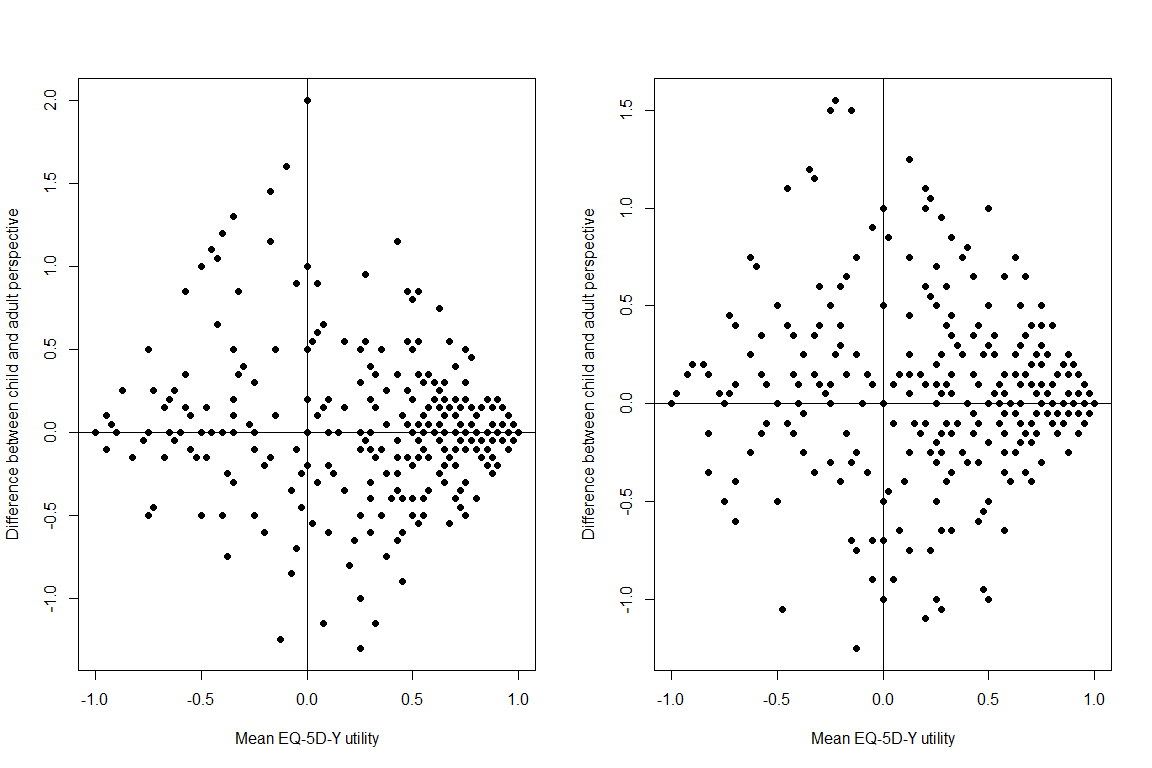


*Figure B4:* Bland-Altman plot (as above) per condition, left: cTTO, right: LT-TTO.

To illustrate the effect of correcting for time preference, we also include the same 4 Figures after correcting for time preference. That is, Figure B5 shows overall distributions for EQ-5D-Y-3L utilities elicited with adult and child perspectives and Figure B6 separates these by condition. The set of Bland-Altman plots are reprinted in Figures B7 and 8. If anything, these pictures illustrate that correcting for time preferences drops censoring of utilities at U(Q)=-1, and thus also the floor effect in the Bland-Altman plot.


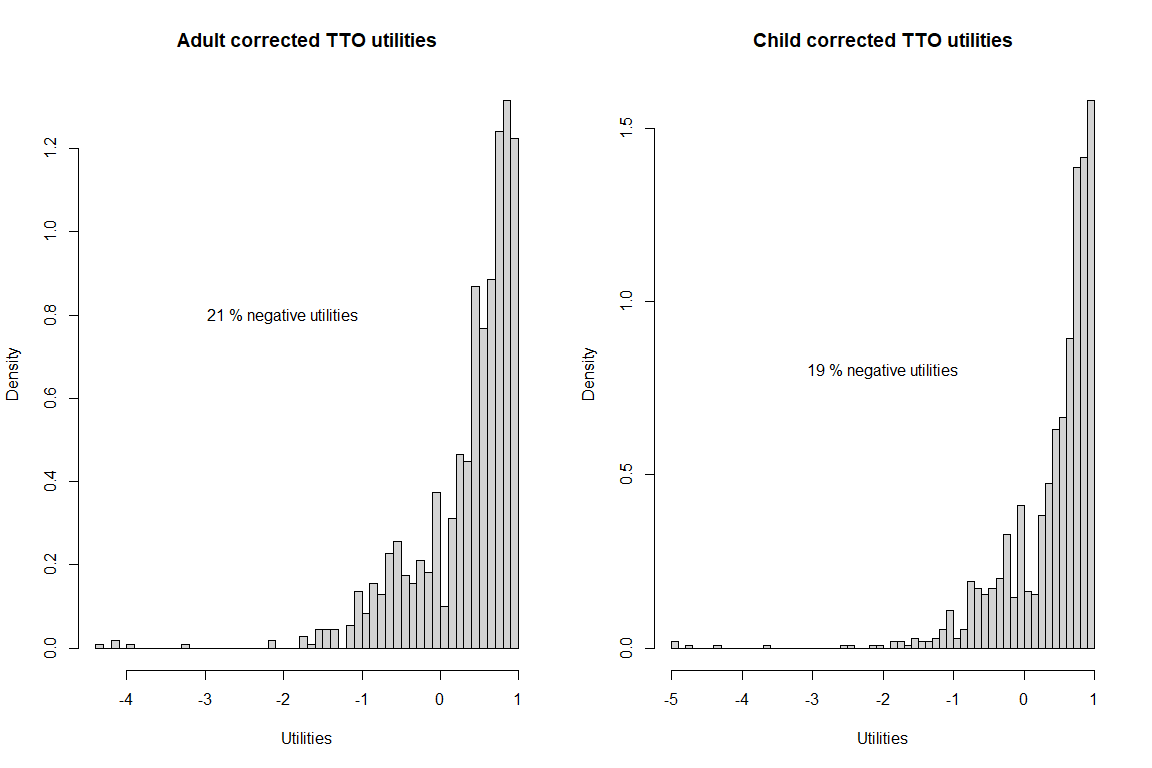


Figure B5: Distribution of **corrected** EQ-5D-Y-3L utilities in adult and child perspectives


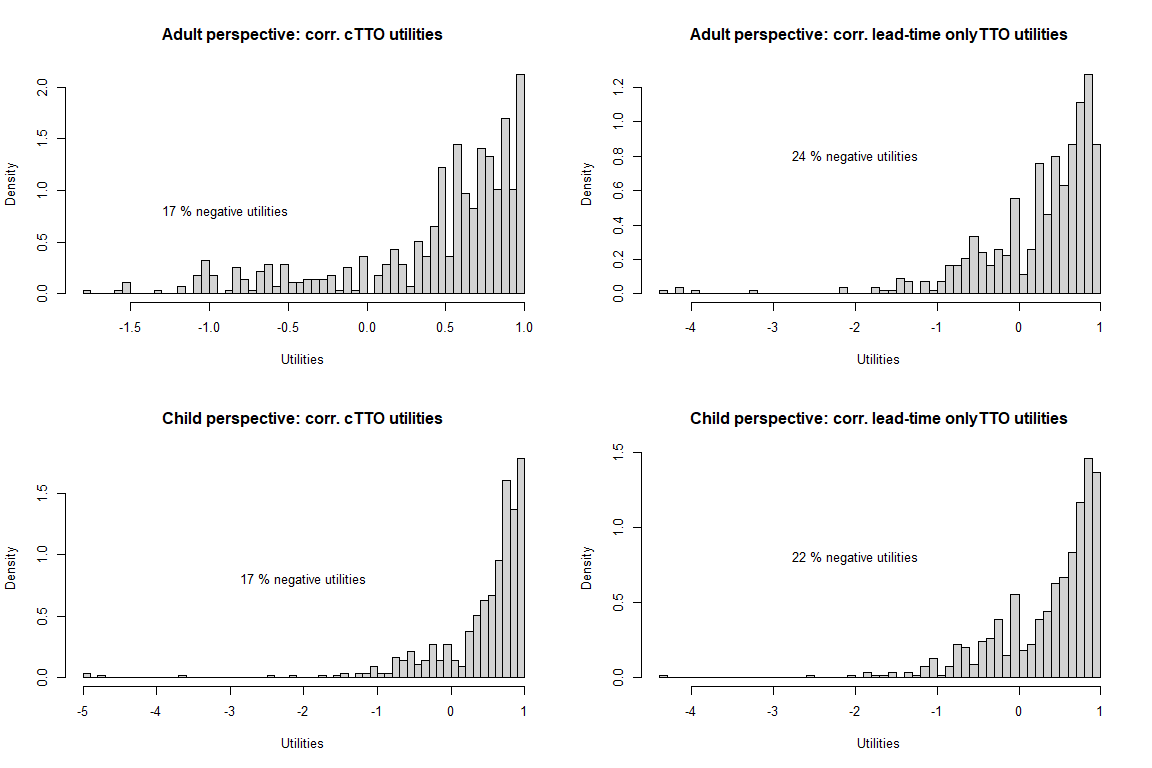


Figure B6: Distribution of **corrected** EQ-5D-Y-3L utilities per condition and perspective


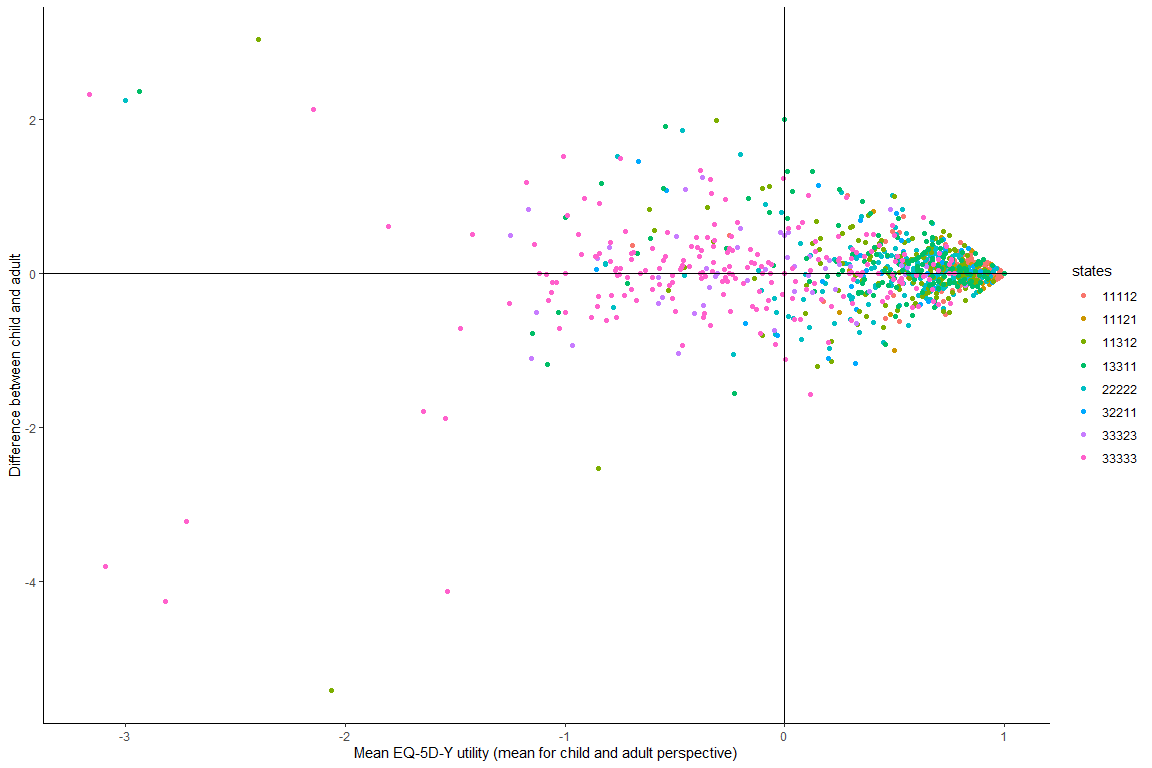


*Figure B7:* Bland-Altman plot with mean **corrected** EQ-5D-Y utility across child and adult perspectives plotted against the difference between the two perspectives.


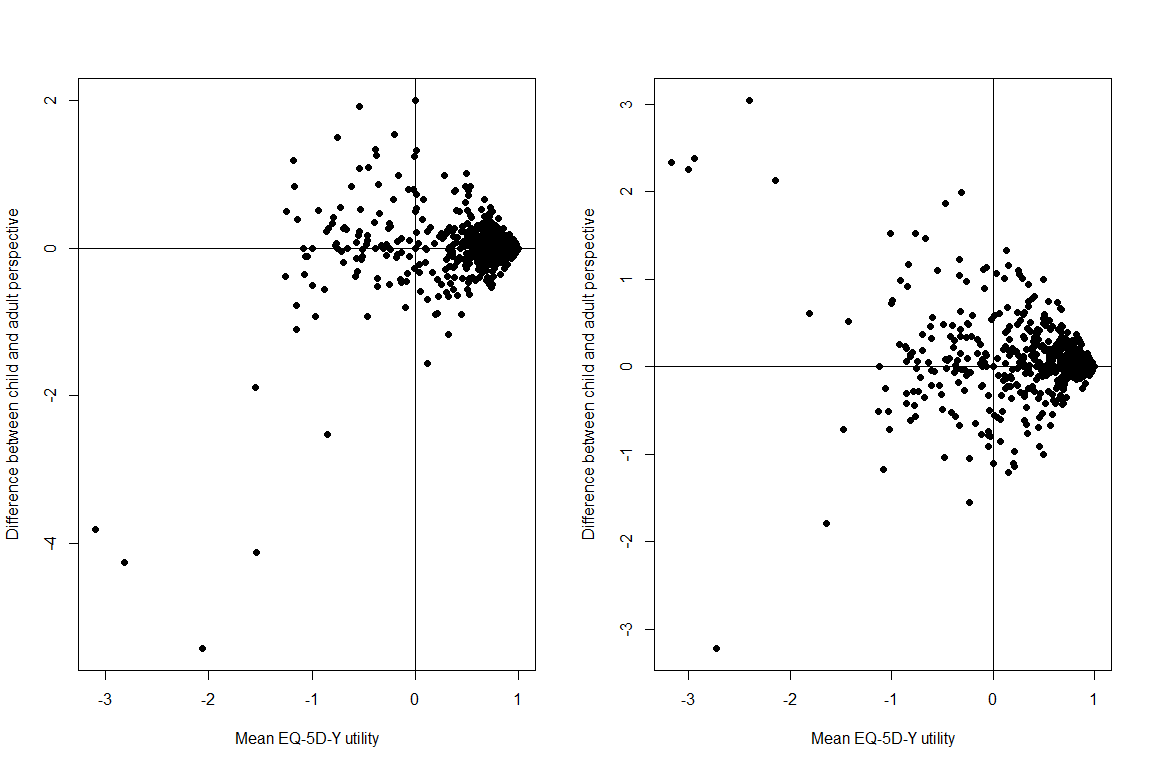


*Figure B8:* Bland-Altman plot (as above) per condition, left: cTTO, right: LT-TTO.

## Appendix C. Additional regression analyses demonstrating the robustness of our finding

This appendix reports the results of an additional set of regression analyses, with a similar model specification as the models reported in Table 5 (i.e. mixed effects models with subject random effects). The analyses reported in Table C1 shows that the effects of perspective and condition are robust to inclusion of:

1. *Payment status (Models 1 and 5):* coded as 0 for course credits and 1 for paid respondents. Payment status does not affect EQ-5D-Y-3L utilities
2. *Order effects (Models 2 and 6):* coded with the following order as reference: direct method first, adult perspective first. These order effects are included in all models except for Model 1 and 5. Note that there is a significant effect of task order, such that utilities are generally higher when TTO valuation was completed *before* time preferences were measured. This appeared to occur regardless of the perspective order.
3. *Age and sex (Models 3 and 7):* where sex is coded with female as the reference category. Although EQ-5D-Y-3L utilities are generally higher among males, the effects of condition and perspective are robust to the inclusion of these demographics.
4. *Other demographics (Models 4 and 8):* The final models include all demographics and task characteristics (i.e. order effects and payment status), as well as a set of self-reported health characteristics. These include self-reported EQ-5D-Y-3L dimensions as well as individuals’ health reported on EQ-VAS.

**Table C1**. Robustness checks with respect of payment method, task order, demographic information before and after time preference correction

|  | Uncorrected | | | | Corrected | | | |
| --- | --- | --- | --- | --- | --- | --- | --- | --- |
|  | (1) | (2) | (3) | (4) | (5) | (6) | (7) | (8) |
| Condition (=0 if cTTO) | -0.116^***^ | -0.130^***^ | -0.130^***^ | -0.128^***^ | -0.121^**^ | -0.135^***^ | -0.133^***^ | -0.134^**^ |
|  | (0.0387) | (0.0373) | (0.0380) | (0.0382) | (0.0515) | (0.0484) | (0.0503) | (0.0528) |
|  |  |  |  |  |  |  |  |  |
| Perspective (=0 if adult) | 0.0482^***^ | 0.0482^***^ | 0.0478^***^ | 0.0478^***^ | 0.0455^**^ | 0.0455^**^ | 0.0451^*^ | 0.0451^*^ |
|  | (0.0144) | (0.0144) | (0.0145) | (0.0145) | (0.0229) | (0.0229) | (0.0231) | (0.0231) |
| *Health state* |  |  |  |  |  |  |  |  |
| 11112 | 0.0103 | 0.00997 | 0.0110 | 0.0125 | 0.0241 | 0.0224 | 0.0241 | 0.0309 |
|  | (0.0434) | (0.0428) | (0.0427) | (0.0445) | (0.0520) | (0.0513) | (0.0514) | (0.0545) |
|  |  |  |  |  |  |  |  |  |
| 22222 | -0.310^***^ | -0.310^***^ | -0.309^***^ | -0.308^***^ | -0.311^***^ | -0.312^***^ | -0.310^***^ | -0.305^***^ |
|  | (0.0397) | (0.0394) | (0.0394) | (0.0404) | (0.0451) | (0.0448) | (0.0449) | (0.0461) |
|  |  |  |  |  |  |  |  |  |
| 32211 | -0.291^***^ | -0.291^***^ | -0.291^***^ | -0.291^***^ | -0.297^***^ | -0.297^***^ | -0.297^***^ | -0.297^***^ |
|  | (0.0477) | (0.0477) | (0.0477) | (0.0477) | (0.0504) | (0.0504) | (0.0504) | (0.0504) |
|  |  |  |  |  |  |  |  |  |
| 11312 | -0.304^***^ | -0.304^***^ | -0.301^***^ | -0.299^***^ | -0.310^***^ | -0.311^***^ | -0.307^***^ | -0.301^***^ |
|  | (0.0437) | (0.0428) | (0.0427) | (0.0447) | (0.0498) | (0.0490) | (0.0489) | (0.0509) |
|  |  |  |  |  |  |  |  |  |
| 33323 | -0.931^***^ | -0.931^***^ | -0.931^***^ | -0.931^***^ | -1.000^***^ | -1.000^***^ | -1.000^***^ | -1.000^***^ |
|  | (0.0658) | (0.0658) | (0.0658) | (0.0658) | (0.0799) | (0.0799) | (0.0799) | (0.0799) |
|  |  |  |  |  |  |  |  |  |
| 13311 | -0.341^***^ | -0.342^***^ | -0.338^***^ | -0.337^***^ | -0.346^***^ | -0.347^***^ | -0.343^***^ | -0.336^***^ |
|  | (0.0465) | (0.0453) | (0.0453) | (0.0472) | (0.0520) | (0.0510) | (0.0510) | (0.0528) |
|  |  |  |  |  |  |  |  |  |
| 33333 | -0.993^***^ | -0.993^***^ | -0.991^***^ | -0.990^***^ | -1.064^***^ | -1.065^***^ | -1.063^***^ | -1.058^***^ |
|  | (0.0456) | (0.0451) | (0.0451) | (0.0463) | (0.0553) | (0.0546) | (0.0546) | (0.0556) |
|  |  |  |  |  |  |  |  |  |
| Paid | 0.0248 |  |  | 0.0436 | 0.00980 |  |  | 0.0222 |
|  | (0.0549) |  |  | (0.0564) | (0.0658) |  |  | (0.0697) |
|  |  |  |  |  |  |  |  |  |
| Taskorder = 2 (TP1st – CA) |  | 0.0533 | 0.0439 | 0.0445 |  | 0.0822 | 0.0696 | 0.0682 |
|  |  | (0.0481) | (0.0496) | (0.0488) |  | (0.0622) | (0.0632) | (0.0619) |
|  |  |  |  |  |  |  |  |  |
| Taskorder = 3 (TTO1st – AC) |  | 0.101^**^ | 0.102^**^ | 0.108^**^ |  | 0.144^**^ | 0.142^**^ | 0.153^***^ |
|  |  | (0.0483) | (0.0482) | (0.0480) |  | (0.0603) | (0.0598) | (0.0593) |
|  |  |  |  |  |  |  |  |  |
| Taskorder = 4 (TTO1st – CA) |  | 0.135^**^ | 0.143^***^ | 0.145^***^ |  | 0.157^**^ | 0.168^**^ | 0.168^**^ |
|  |  | (0.0550) | (0.0550) | (0.0533) |  | (0.0801) | (0.0791) | (0.0811) |
|  |  |  |  |  |  |  |  |  |
| Gender (=0 if female) |  |  | 0.0701^*^ | 0.0714^*^ |  |  | 0.0959^**^ | 0.101^**^ |
|  |  |  | (0.0386) | (0.0393) |  |  | (0.0455) | (0.0466) |
|  |  |  |  |  |  |  |  |  |
| Age |  |  | -0.00715 | -0.00879 |  |  | -0.00663 | -0.00807 |
|  |  |  | (0.00644) | (0.00618) |  |  | (0.00744) | (0.00721) |
|  |  |  |  |  |  |  |  |  |
| Other controls |  |  |  | X |  |  |  | X |
| *N* | 2190 | 2190 | 2170 | 2170 | 2190 | 2190 | 2170 | 2170 |

Standard errors in parentheses. All standard errors are clustered at the individual level.

^*^ *p* < 0.10, ^**^ *p* < 0.05, ^***^ *p* < 0.01

Note: We have eight health states in total, here we take the mild state 11121 as the reference when interpreting the change of the TTO utility for each health state. *Paid* =1 represents that the subjects are rewarded by 7 euros for this experiment, the others are rewarded by course credit. Other controls include the experiment duration, self EQ-VAS health rating and EQ-5D five dimensions (mobility, self-care, usual activities, pain/discomfort and anxiety/depression). "X" indicates that we control these variables in that column estimation. We have 219 subjects in total, each subject needs to answer five health states utilities in both own perspective and child's perspective. For columns (3)(4)(7)(8) when including the age, there are two subjects failing to report the age information.
